# Impact of a microfluidic jet on a pendant droplet†

**DOI:** 10.1039/d1sm00706h

**Published:** 2021-06-28

**Authors:** Miguel A. Quetzeri-Santiago, Ian W. Hunter, Devaraj van der Meer, David Fernandez Rivas

**Affiliations:** Mesoscale Chemical Systems Group, MESA+ Institute and Faculty of Science and Technology, University of Twente P. O. Box 217 7500AE Enschede The Netherlands m.a.quetzerisantiago@utwente.nl d.fernandezrivas@utwente.nl; BioInstrumentation Laboratory, Department of Mechanical Engineering, Massachusetts Institute of Technology Cambridge Massachusetts 02139 USA; Physics of Fluids group and Max Plank Center Twente, Mesa + Institute and Faculty of Science and Technology, J. M. Burgers Centre for Fluid Dynamics and Max Plank Center Twente for Complex Fluid Dynamics, University of Twente P. O. Box 217, 7500AE Enschede The Netherlands

## Abstract

High speed microfluidic jets can be generated by a thermocavitation process: from the evaporation of the liquid inside a microfluidic channel, a rapidly expanding bubble is formed and generates a jet through a flow focusing effect. Here, we study the impact and traversing of such jets on a pendant liquid droplet. Upon impact, an expanding cavity is created, and, above a critical impact velocity, the jet traverses the entire droplet. We predict the critical traversing velocity (i) from a simple energy balance and (ii) by comparing the Young–Laplace and dynamic pressures in the cavity that is created during the impact. We contrast the model predictions against experiments, in which we vary the liquid properties of the pendant droplet and find good agreement. In addition, we assess how surfactants and viscoelastic effects influence the critical impact velocity. Our results increase the knowledge of the jet interaction with materials of well-known physical properties.

## Abstract 2

The impact of microscopic jets on pendant liquid droplets is studied experimentally. The critical traversing jet velocity is predicted, which explains how the phenomena are governed by the impact inertia, the surface tension of the droplet and viscoelastic effects.

## Graphical abstract

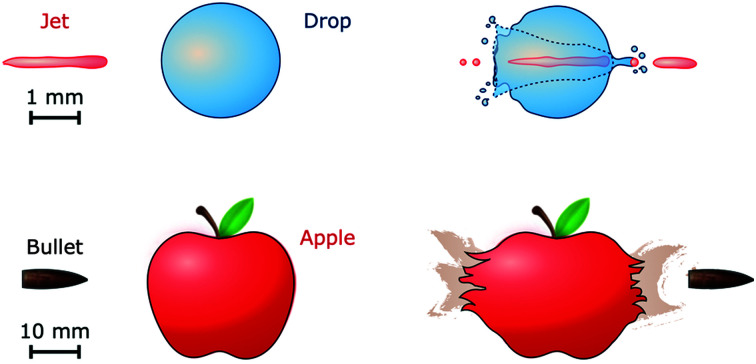

## 1 Introduction

The impact of a solid or liquid object into a deep liquid pool generates a cavity with dynamics first described by Worthington in 1908.^1^ Since then, research has focused on many topics, including the critical energy necessary for air entrainment into a pool, the collapse of the formed cavity, and the subsequent formation of Worthington jets.^2–7^ The projectiles studied in the literature usually have sizes in the range of 1 to 5 mm, an impact speed range of 1 to 10 m s^−1^, and the pool is usually orders of magnitude larger than the projectile and the created cavity.^2–7^ In these cases, hydrostatic pressure has been found to be a major driver for the collapse and retraction of the cavity made on the liquid pool.^2^ In contrast, we found just two works discussing the impact of projectiles in the submillimeter range.^8,9^ In this paper we study for the first time the impact of micrometer-sized jets (≈100 μm) on a self-contained liquid object, namely droplets of ≈2 mm. In this case, the jets travelling at speeds of ≈20 m s^−1^ generate a cavity circumscribed by the droplet volume and the hydrostatic pressure can be neglected.

**Fig. 1:**
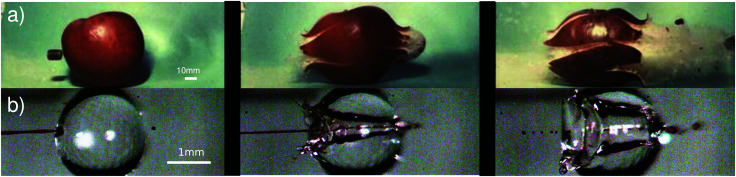
Comparison between a sequence of images from, (a) Harold E. Edgerton, Bullet through Apple, 1964, *U*_bullet_ ∼ 500 m s^−1^ (reprinted with the permission of James W. Bales and Andrew Davidhazy). The sequence is taken using the flash photography technique,^10^ with a flash duration of ≈1/3 μs. The flash is triggered by an electronic circuit that reacts to the sound of a rifle shot. (b) The impact of a liquid jet on a droplet; the video is recorded using a high-speed camera at 50k frames per second, and the jet diameter is *D*_jet_ = 100 m and its impact velocity is *U*_jet_ = 25.8 m s^−1^ (see also Movie S1 in the ESI†). Apart from the striking aesthetic resemblance of the processes, the ratio between the projectile kinetic energy and the energy associated with the target's resistance to deformation is on the same order of magnitude, see Section 3.1.

Since the dynamics of the aforementioned events take place in a few milliseconds, high-speed imaging is required to observe the phenomena. High-speed imaging was pioneered by scientists like Harold E. Edgerton, using his strobe-flash photography technique.^10^ One of his most famous sequences of pictures is that of a 0.22 inch caliber bullet traversing an apple at ≈380 m s^−1^ (Fig. 1a). In this sequence, the apple is opaque, but, what if we could replace the target with a transparent object, *i.e.*, a liquid apple? This is precisely the case when we target a droplet with a liquid microjet. As shown in Fig. 1, the aesthetics of a high-speed jet traversing a liquid droplet and Edgerton's pictures are strikingly similar. The difference is that, with a translucent liquid, we can observe the impact dynamics inside the droplet, and besides producing impressive images, high-speed imaging facilitates the description of the fast phenomena, such as providing the projectile speed and the target deformation.

Our aim in this paper is to unravel the physics that governs the impact of liquid water jets on a pendant droplet of liquids with different properties. This knowledge increases our understanding of the jet interaction with materials of well-known physical properties. Such an understanding can advance our knowledge on needle-free injections, because jets of similar sizes and speeds in the present study have been proposed for that purpose.^11–13^

The paper is structured as follows. The experimental procedure is outlined in Section 2. In Section 3 we present two models to predict the critical jet velocity for traversing a droplet: in Section 3.1 we use an energy balance between the kinetic energy of the jet and the surface energy of the droplet. In Section 3.2, we employ the two-dimensional Rayleigh equation to obtain the cavity shape and combine it with the Young–Laplace equation to predict its collapse. Next, the model prediction is contrasted against experimental results in Section 4.1. Additionally, in Sections 4.2 and 4.3 we present experimental results on the cavity advancing and retracting velocities with some observations on what occurs after the cavity collapse.

## 2 Experimental method

High-speed jets were generated *via* a thermocavitation process and directed to impact a pendant droplet of different liquids with varying properties. Thermocavitation refers to the phenomenon in which a liquid is vaporised locally by means of a focused laser, leading to bubble nucleation.^14,15^ The expansion of the nucleated bubble can be controlled on a microfluidic chip to generate a jet through a flow-focusing effect.^16,17^ These jets may reach speeds of the order of 100 m s^−1^, which is enough to pierce the skin, and has potential for the transdermal delivery of a liquid, *e.g.* needle-free injections.^18,19^ However, in the experiments described here, we restrict the jet velocity *U*_jet_ to the range of 8–35 m s^−1^, which is sufficient for a jet to traverse droplets of the liquids studied. Additionally, the diameter *D*_jet_ of the liquid jet was in the range of 50–120 μm. Both *U*_jet_ and *D*_jet_ were controlled by varying the laser spot size and power.

**Fig. 2:**
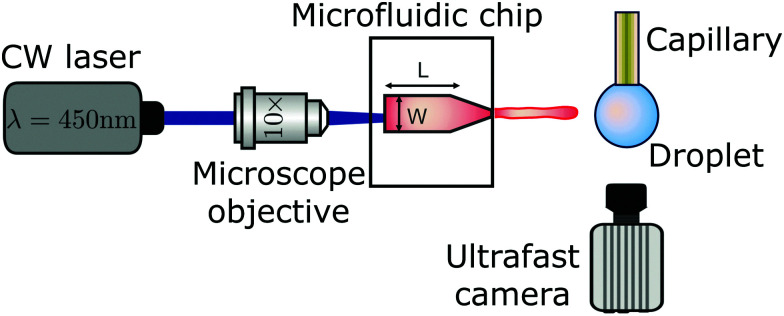
Schematic of the experimental setup. Thermocavitation is obtained focusing a CW laser on the bottom of a microfluidic device with a microscope objective. The thermocavitated bubble expands and creates a liquid jet that is directed at a pendant droplet. The process is recorded with a high-speed camera with the illumination coming from a light source from the front so the cavity evolution can be observed.

The experimental setup is shown in Fig. 2. A Borofloat glass microfluidic chip fabricated under cleanroom conditions is filled with a water solution containing a red dye (Direct Red 81, CAS No. 2610-11-9) at 0.5 wt%. The red dye enhances the laser energy absorption and facilitates bubble nucleation. The microfluidic device has a tapered channel with an angle *α* = 15 degrees to avoid swirling of the jet,^17^ nozzle diameter *d* = 120 μm, channel length *L* = 1050 μm and width *W* = 600 μm. The thermocavitation bubble is created by focusing a continuous wave laser diode (Roithner LaserTechnik, wavelength *Λ* = 450 nm and nominal power of 3.5 W), on the microchannel using a 10× microscope objective. The liquids used were water, ethanol, aqueous solutions of glycerol, Triton X-100 and sodium-bis(2-ethylhexyl)sulfosuccinate (Aerosol OT) at different concentrations and polyethylene-oxide of varied molecular weights (PEO). Liquid droplets were created by holding a capillary tube with an outer diameter of 360 μm, controlling the volume with a precision glass syringe and a syringe pump (Harvard PHD 22/2000). All chemical additives were bought from Sigma-Aldrich. The properties of the Newtonian and non-Newtonian liquids used are reported in Table 1. The surface tension of all the liquids was measured with Pendent Drop ImageJ plugin,^20^ and their shear viscosity was measured with an Anton Paar MCR 502 rheometer.

**Table 1:** List of fluids used providing their shear viscosity *μ*, surface tension *γ* and density *ρ*. The viscoelastic relaxation time *λ* is also shown for the polyethylene-oxide solutions

Fluid	*μ* (mPa s)	*γ* (mN m^−1^)	*ρ* (kg m^−3^)	*λ* (ms)
Ethanol	1.04	26.3	789	—
Water	1.0	72.1	998	—
Aqueous glycerol 25 v%	2.4	69.7	1071	—
Aqueous glycerol 50 v%	8.4	67.6	1142	—
Aqueous glycerol 70 v%	28.7	66.1	1193	—
Aqueous glycerol 78 v%	43.6	65.2	1212	—
Triton 0.2 CMC%	1.0	43.9	998	—
Triton 1 CMC%	1.0	30.8	998	—
Triton 3 CMC%	1.0	32.5	998	—
Aerosol OT 1 wt% (AOT 1%)	1.0	23.4	998	—
Aerosol OT 0.1 wt% (AOT 0.1%)	1.0	24.1	998	—
Water & red dye 0.5 wt%	0.91	47.0	1000	—
PEO 100k 0.1 wt%	1.03	63.2	996	0.006
PEO 100k 1 wt%	2.43	62.9	995	0.047
PEO 100k 10 wt%	50.8	62.5	1001	0.333
PEO 600k 0.1 wt%	1.56	63.1	996	0.307
PEO 600k 1 wt%	21.7	62.9	998	1.317

**Fig. 3:**
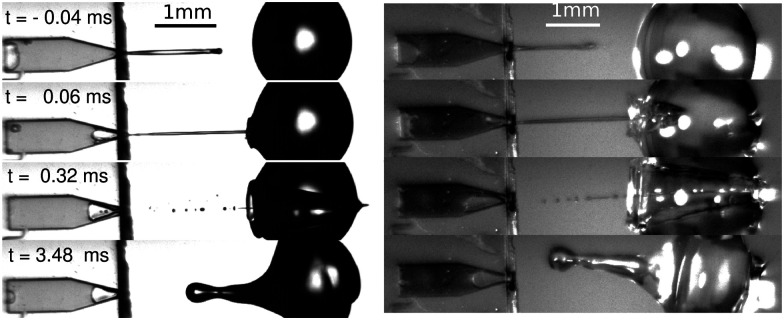
Snapshot sequences of a jet impacting on a water droplet with Triton X-100 at 3 CMC (left, Movie S2 in the ESI†) and a pure water droplet (right, Movie S3 in the ESI†). In the former *U*_jet_ = 16.3 m s^−1^, We_jet_ = 654 and *D*_drop_ = 1.65 mm. In the latter *U*_jet_ = 17.1 m s^−1^, We_jet_ = 325 and *D*_drop_ = 1.86 mm. The imaging of the left sequence is done with a conventional shadowgraph illumination system. In contrast, the right sequence is taken with a front light illumination system, where the cavity dynamics can be more clearly observed. Times are taken relative to the impact moment at *t* = 0. The impact process is qualitatively the same for both of the droplets; a cavity is generated inside the droplet (*t* ∼ 0.06 ms), the jet traverses the droplet (*t* ∼ 0.032 ms) and a rebound Worthington jet is generated (*t* ∼ 3.48 ms). Time is taken from the impact moment *t* = 0 ms.

The processes of bubble generation, jet ejection and impact on the liquid droplet were recorded using a Photron Fastcam SAX coupled with a 2× microscope objective. A typical experiment duration was ∼5 ms and the camera resolution was set to 768 × 328 pixels^2^ at a sample rate of 50k frames per second with an exposure time of 2.5 μs. Typical images obtained from the experiments are shown in Fig. 3, where one can observe how a water droplet is pierced by the liquid jet produced from the microchip on the left, using shadowgraph imaging (left) and direct lighting (right). Experiments were carried out with a typical shadowgraph configuration, and we switched to a front light illumination system to observe the cavity dynamics. In the front illumination system a white background was placed to enhance the image contrast and increase the light reaching the camera sensor. Image analysis to extract the jet diameter, impact velocity and cavity dynamics was performed with a custom generated MATLAB script. The shadowgraph imaging benefits from more light reaching the sensor, and thus a smaller camera exposure time can be used, leading to a better jet definition. However, extracting information from the expanding cavity is impossible. In contrast, with front light imaging we can extract the information from the expanding cavity, but the jet is not as well defined as that using shadowgraphy.

## 3 Critical jet velocity

In this section, we predict the critical velocity needed for a jet to traverse a droplet using two different approaches: (i) by using a simple energy balance and (ii) by comparing the Young–Laplace and dynamic pressures in the cavity that is created during impact. In Section 3.1, we start from an energy analysis of Edgerton's experiment of a bullet traversing an apple and subsequently transfer the argument to the droplet case of our current study. With this example we introduce the concept of kinetic energy of the projectile and the resistance of the target to being traversed. Moreover, we deduce the critical velocity of the jet by doing an energy balance between the kinetic energy of the jet and the surface energy of the droplet. Additionally, in Section 3.2, we use Rayleigh's two-dimensional equation to predict the shape of the cavity and predict its collapse with the Young–Laplace equation, thus finding the jet critical traversing velocity.

### 3.1 Energy balance between the jet kinetic energy and the droplet surface energy

In his lecture titled How to Make Applesauce at MIT, Edgerton presented his famous series of pictures of bullets traversing apples presented in Fig. 1a. This set of images illustrated the traversing process, but did not reflect on the energy of the bullet or the energy of the apple. What would it take the apple to stop the bullet? Or equivalently, what would be the necessary speed for the bullet to get trapped and embedded inside the apple?

In this section, we will answer these questions by using an energy balance between the kinetic energy of a bullet *E*_*k*_bullet__ = *M*_bullet_*U*_bullet_^2^/2, where *M*_bullet_ is the mass of the bullet, and the toughness of an apple *T*_apple_, which we define as its ability to absorb energy by elastoplastic deformation without fracturing. Hence, by performing the energy balance, the critical velocity for the bullet to penetrate the apple may be written as1
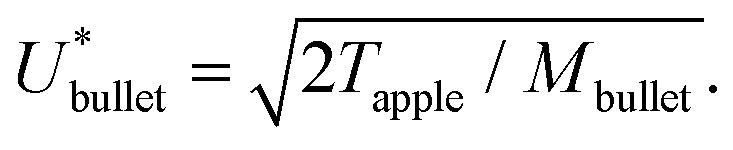


Since we considered that the whole apple absorbs all the energy of the bullet impact, we remark that the energy balance is valid just when the diameter of the bullet has a similar size to that of the apple. The mass of a 0.22 caliber bullet is *M*_bullet_ ≈ 10 g and the apple toughness is *T*_apple_ ≈ 10 J.^21^ Therefore, 
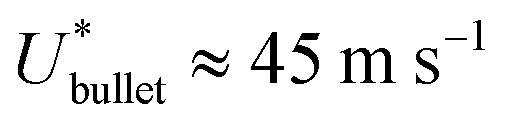
, which is at least one order of magnitude smaller than the typical velocities reached by 0.22 caliber bullets, *U*_bullet_ ≈ 380 m s^−1^.^22^ Consequently, it is understandable that the apple is traversed by the bullet in Edgerton's photographs.

**Fig. 4:**
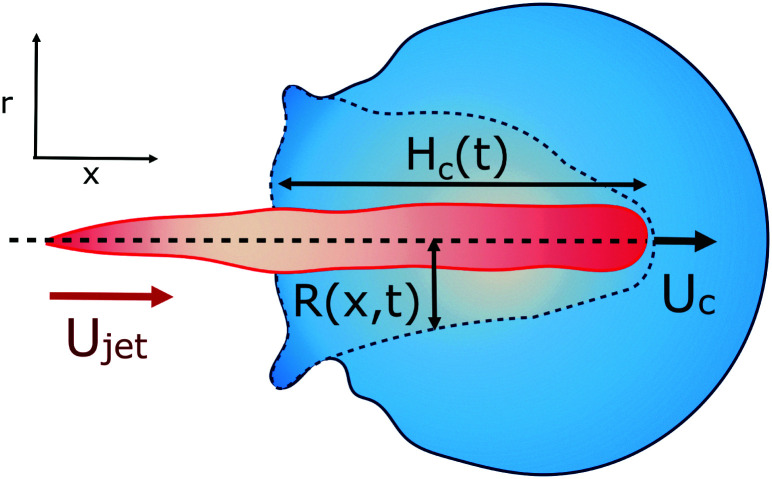
Diagram illustrating the parameters used in this section. The jet impacts the pendant droplet from the left with velocity *U*_jet_, creating a cavity with increasing depth in time *H*_c_(*t*), where the velocity of the apex of the cavity is indicated by *U*_c_ = *Ḣ*_c_ and whose radius depends on time and the position *R*(*x*, *t*). Here, *x* is the direction along which the jet travels and *r* is its perpendicular radial direction.

For our liquid jet, the kinetic energy is *E*_*k*jet_ ≈ (π/8)*ρ*_jet_*U*_jet_^2^*D*_jet_^2^*H*_jet_, with *ρ*_jet_ and *H*_jet_ being the density and length of the jet, respectively, and the resisting force of the droplet is dominated by its surface energy. For the critical conditions where the jet traverses the droplet, the jet kinetic energy transforms into the surface energy of the cavity generated at impact. For simplicity, assuming that the cavity geometry is cylindrical, the cavity surface energy is *E*_*γ*c_ ≈ π*D*_c_*D*_drop_*γ*_drop_, with *γ*_drop_ being the droplet surface tension and *D*_c_ being the cavity diameter. Here, *D*_c_ is constrained by *D*_drop_ and as shown in Fig. 3 and 4, *D*_c_ ∼ *D*_drop_. Also, since the velocity of the tip of the cavity is approximately half the jet velocity 
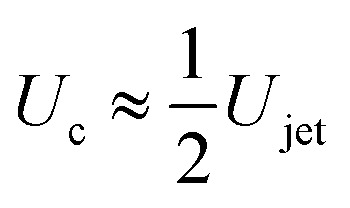
, the total length of the jet would not contribute to the traversing process but only a part of it, namely 
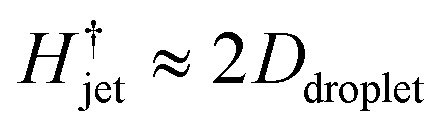
.^8^ Using this limiting value 
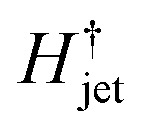
, the jet critical velocity for droplet traversing is2
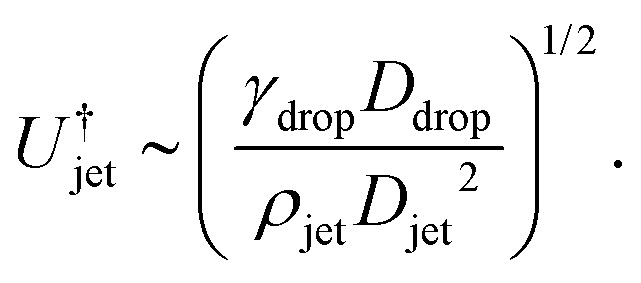
Defining the relevant Weber number of the jet as We_jet_ = *ρ*_jet_*U*_jet_^2^*D*_jet_/*γ*_drop_, and substituting in eqn (2) we find the critical minimal Weber number needed to traverse the droplet3
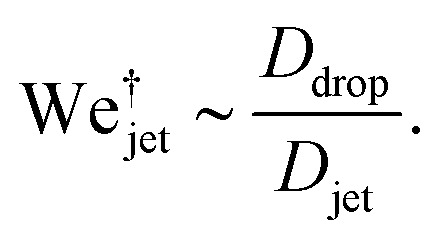
Substituting typical values of a jet impacting a water droplet in our experiments (*ρ*_jet_ ≈ 1000 kg m^−3^, *D*_jet_ ≈ 100 μm, *D*_drop_ ≈ 2 mm and *γ*_drop_ ≈ 0.07 mN m^−1^), we obtain *U*^†^_jet_ ≈ 4 m s^−1^ or We^†^ ≈ 20.

Now, we have all the ingredients to do a scaling comparison between a bullet traversing an apple and a jet traversing a droplet. Taking the values of *U*_jet_ and *U*_bullet_ from the experiments in Fig. 1a and b (which are well above the critical value for penetration in both cases) and the target toughness (toughness for an apple and surface energy for a droplet), we get *E*_*k*_bullet__/*T*_apple_ ∼ *E*_*k*_jet__/*E*_*γ*_c__ ∼ 100. Therefore, the relative energies involved in both processes are of the same order of magnitude, indicating that the traversing phenomena in both cases share more than aesthetic similarities. As both processes are in a regime dominated by inertia, we expect that the cavity creation follows similar physics. Indeed, in the context of shaped charges, the penetration of a solid onto another solid has been described from the fluid dynamics perspective.^23^ Nevertheless, after impact, the apple fractures and does not possess the restoring force a liquid droplet has, namely, the surface tension. This leads to a large discharge of mass at the back of the apple, with a cavity bigger at the exit point than at the entry point. In contrast, the droplet can redistribute its mass without breaking and the surface cavity closes due to the surface tension. This is the cause of the much appreciated fact that we did not have to deal with substantial amounts of debris after our experiments.

### 3.2 Comparison between the Young–Laplace and dynamic pressures of the cavity

Considering the mass of a cylindrical liquid jet with radius *R*_jet_ and length *H*_jet_ falling into a pool of the same liquid, air is entrained in the pool at sufficiently energetic impacts, *i.e.*, We ≫ 1 and Re ≫ 1.^2^ Additionally, the cavity dynamics and the air entrainment depend on the aspect ratio of the jet. The limiting cases are *H*_jet_/*R*_jet_ → ∞, corresponding to the impact of a continuous jet, and *H*_jet_/*R*_jet_ → 1, where the case of a droplet impact onto a liquid pool is recovered.^2,24^ For the former case, the apex of the cavity advances with a velocity 
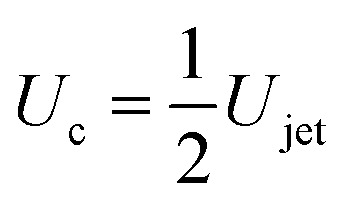
, therefore, the depth of the cavity can be estimated as 
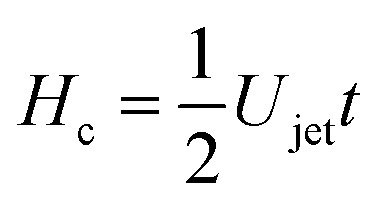
.^2,8,9^

In the cavity formation of a droplet impacting a liquid surface, the process is mainly inertial during the first instances, with surface tension becoming important at the moment near the maximum depth of the cavity *H*_max_.^8^ Additionally, Deng *et al.* in 2007^25^ showed that viscous dissipation accounts for ∼1.4% of the initial kinetic energy loss of a water droplet of *D* = 2.5 mm impacting a liquid pool. Therefore, assuming that the cavity shape is slender and the process is inertia dominated, *i.e.*, neglecting viscous dissipation, we can apply the two-dimensional Rayleigh equation in cylindrical coordinates to predict the cavity shape,^26,27^4
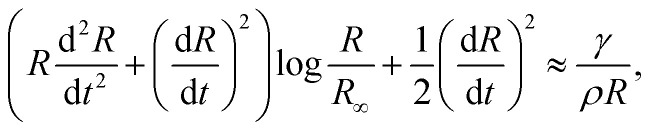
where *R*(*x*, *t*) is the radius of the cavity, *x* is the position of the cavity in the horizontal direction and *R*_∞_ is an external length scale (see Fig. 4). Following the argument of Bouwhuis *et al.*,^8^ during the first instances of the cavity formation, inertia dominates and the dynamics are determined by *R*(d^2^*R*/d*t*^2^) + (d*R*/d*t*)^2^ ≈ 0. Solving this equation we get *R*(*t*) ∼ (*t* − *t*_0_)^1/2^, where *t* = *H*_c_/*U*_c_ and *t*_0_ = *x*/*H*_c_, and the approximate cavity profile is,^8^5
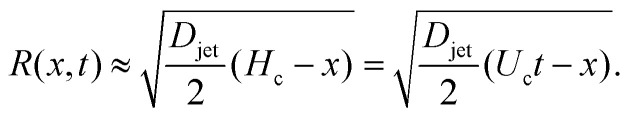
The time *t*_c_ at which surface tension can influence the cavity walls can be predicted by comparing the dynamic pressure of the radially expanding cavity and the Young–Laplace pressure based on the azimuthal curvature of the cavity,6
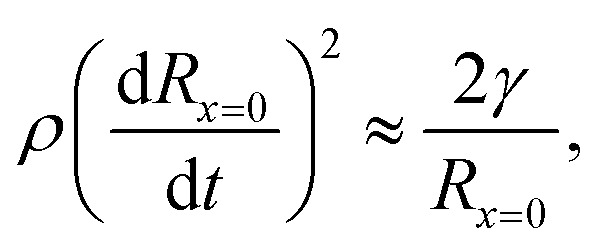
where *R*_*x*=0_ = *R*(0, *t*) is the cavity radius at the jet impact point *x* = 0. Taking the cavity profile from eqn (5), we get 
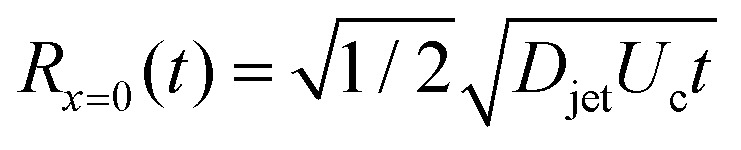
, and 
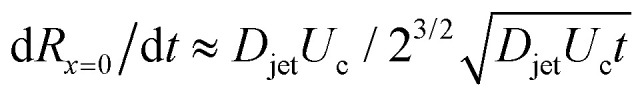
.^8^ Therefore,7
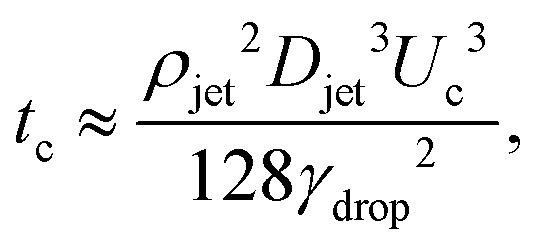
and

8 *H*_max_ ≈ *U*_c_*t*_c_.

The condition for the jet to traverse the droplet is *D*_drop_ < *H*_max_. Using 
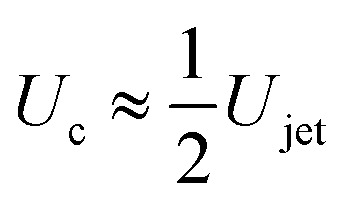
 and eqn (7) and (8), the critical impact velocity for the jet to traverse the droplet is9
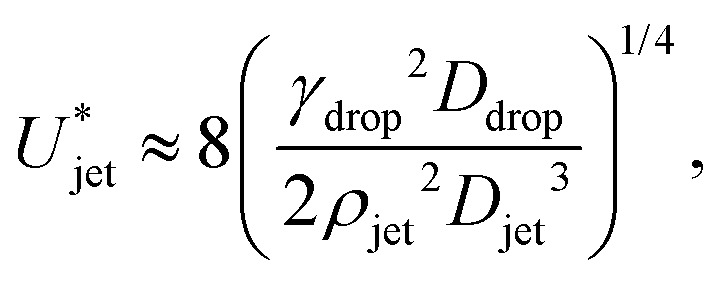
and10
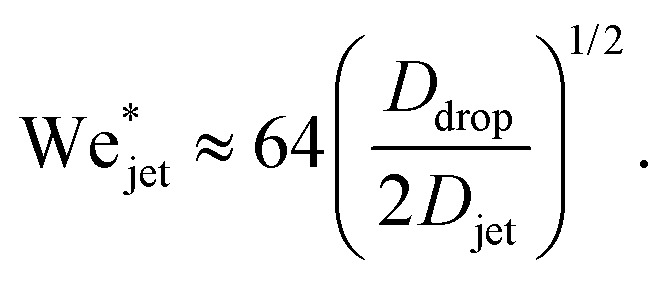
For a jet impacting a water droplet, *D*_drop_ = 2 mm, *γ*_drop_ = 0.072 N m^−1^, *D*_jet_ = 100 μm and *ρ*_jet_ = 1000 kg m^−3^, we observe that the critical velocity needed to traverse the droplet is 
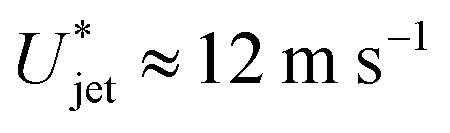
, which is three times larger than 
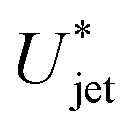
 obtained from eqn (2). Similarly, 
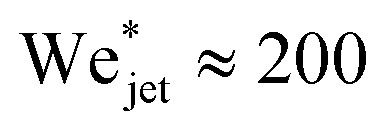
 which is about ten times as large as for eqn (3).

While the results in eqn (3) and (10) are of the same order of magnitude, their dependence on the *D*_drop_/*D*_jet_ ratio is different, namely linear in eqn (3) whereas in eqn (10), there is a square root dependence. This discrepancy arises from the difference in the geometric shape of the generated cavity that was assumed in the two approaches, resulting in a different surface energy. Indeed, a very simple cylindrical geometry was assumed during the energy balance method. In contrast, deriving eqn (10) using the Rayleigh equation leads to a more rigorous description of the cavity shape. Therefore, we consider the latter model to be more accurate and in the following section will compare our experimental data to eqn (10).

## 4 Results and discussion

In this section we will describe our experiments on the traversing of the jet through the droplet and compare them to the above criterion. Furthermore, we modify the criterion to include the concept of dynamic surface tension of the droplet *γ*_dyn_ in the case of surfactant covered droplets. After that we will briefly discuss the cavity dynamics, focusing on the motion of the apex of the cavity inside the droplet. Finally, we comment on our observations for droplets containing surfactants and non-Newtonian liquids.

### 4.1 Critical velocity for traversing

**Fig. 5:**
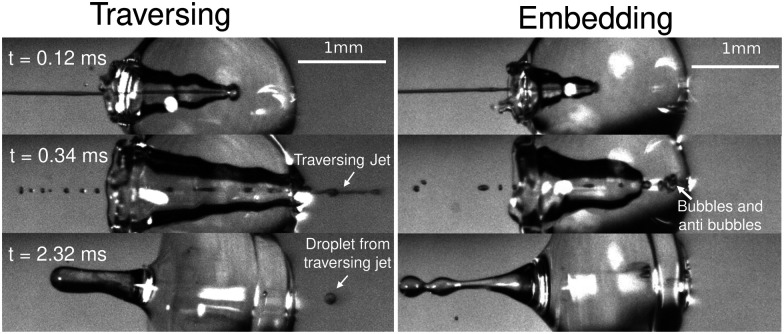
Left, snapshots of a liquid jet impacting (We_jet_ = 587) and traversing a PEO 100k 0.1 wt% droplet (*D*_drop_ = 2.09 mm). Here, we observe that the jet continues its trajectory even after going through all the droplet. Right, snapshots of a liquid jet impacting (We_jet_ = 362) and getting embedded in a PEO 100k 0.1 wt% droplet (*D*_drop_ = 2.08 mm). During embedding, bubbles and antibubbles can be created, see Movie S4 in the ESI.† We note that in both image sequences a rebounding Worthington jet is observed at *t* = 2.32 ms.

We start the discussion of our experimental results by making a qualitative description of the observed phenomena. Fig. 5 shows an image sequence from two typical experiments. Upon impact of the jet on the droplet, a cavity is generated inside the droplet. The increase in the diameter and depth of the cavity with time and its growth rate are dependent on the impact conditions.^9^ At a velocity above a critical value, the jet traverses the droplet completely, as is observed in the left panel of Fig. 5. In contrast, if the jet velocity is not sufficiently large, the jet gets embedded in the droplet and bubbles and anti-bubbles may be created, as in the right panel of Fig. 5 (see also Song *et al.* 2020^28^). Finally, and irrespective of which of these two scenarios applies a rebound Worthington jet is generated.

Now we move on to verifying the validity of the traversing criterion expressed in the critical Weber number obtained in eqn (10), for varying droplet properties. To compare the experimental data and the model presented in Section 3.2, we use the ratio between the experimentally obtained Weber number We_jet_ and the expected critical Weber number 
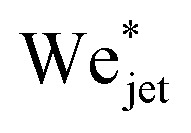
 from eqn (10). Additionally, for the droplets that contain surfactants we need to take into account the fact that, when the jet impacts the droplet and the cavity starts to form, new surface area is created and the surface density of the surfactant decreases. Therefore, the surface tension locally increases from the surface tension measured at equilibrium and the cavity presents a dynamic surface tension *γ*_dyn_.^29^ Consequently, in the surfactant case we re-define the Weber number as 
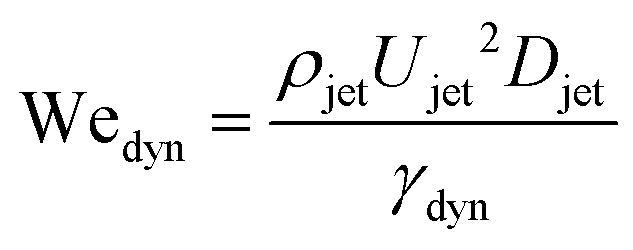
, *i.e.*, using *γ*_dyn_ in its definition, and divide it by the critical value 
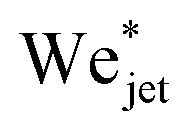
 leading to,11
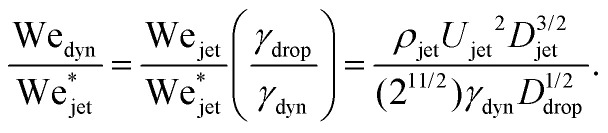


Clearly in the above equation, for the droplets that do not contain surfactants (the glycerol solutions, water and ethanol) we just insert *γ*_dyn_ = *γ*_drop_. For Triton X-100 solutions, the dynamic surface tension can be assessed by the diffusion scale *T*_D_, which is the time required for the surface tension to decrease from the surface tension of water to the equilibrium surface tension.^30^ The diffusion scale depends on the diffusion coefficient of the surfactant (for Triton X-100 *δ* = 2.6 × 10^−10^ m^2^ s^−1^), the maximum surface concentration of the surfactant (*Γ* = 2.9 × 10^−6^ mol m^−2^), the Langmuir equilibrium adsorption constant (*K* = 1.5 × 10^3^ m^3^ mol^−1^) and its volume concentration *C*.^30,31^ For the 3 CMC Triton X-100 solution (the largest concentration used in these experiments), *T*_D_ ∼ 70 ms, while the characteristic timescale of the traversing/embedding process is ∼0.5 ms, *i.e.*, two orders of magnitude smaller. Hence, the dynamic surface tension does not have enough time to reach the measured equilibrium surface tension. Therefore, we do not expect the equilibrium surface tension of Triton X-100 solutions to be relevant in the jet traversing process. Consequently, we assume the dynamic surface tension *γ*_dyn_ of the Triton X-100 solutions to be that of water.

In contrast, AOT being a vesicle surfactant can migrate faster than micelle surfactants such as Triton X-100.^32,33^ In addition, it was shown that at ∼10 ms the dynamic surface tension of an AOT solution at 1 wt% can decrease to a value of ∼32 mN m^−1^.^32^ Therefore, we assume that *γ*_dyn_ for the AOT solutions is ∼32 mN m^−1^. We should note, however, that AOT dynamics are more complex than those of Triton, and characterisation using a single time scale is an oversimplification.

Fig. 5 shows the experimental results of traversing and embedding impact cases as a phase diagram, where on the vertical axis we plot the ratio of the (dynamic) Weber number We_dyn_ and the expected critical Weber number 
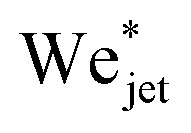
, using eqn (11) such that based upon the model described in Section 3.2 we would expect a transition at 
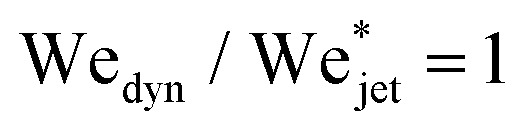
. On the horizontal axis we separate the liquid properties of the droplet by plotting the Ohnesorge number, defined as 
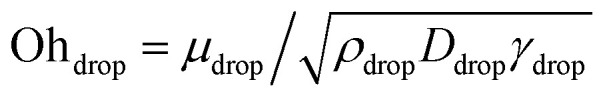
, which is the ratio between viscous forces to inertial and surface tension forces, and has the advantage that is a material property, *i.e.*, it is independent of the dynamics. Open symbols in Fig. 5 represent cases where the jet was observed to traverse the droplet (as in Fig. 5, left) and solid symbols represent the situation where the jet does not traverse the droplet, *i.e.*, becomes embedded as seen in Fig. 5, right.

**Fig. 6:**
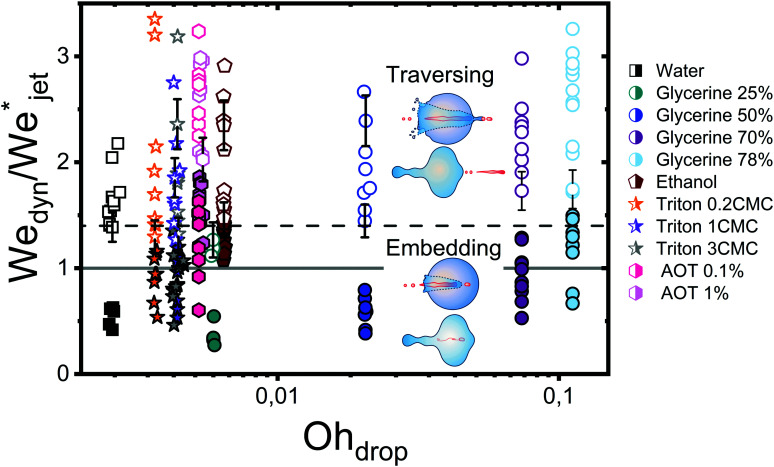
Phase diagram with the rescaled Weber number 
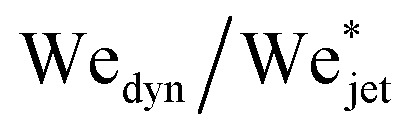
 on the vertical axis and the Ohnesorge number Oh_drop_ on the horizontal axis. Open symbols represent cases where the jet traverses the droplet, while solid ones stand for the embedding case. The grey line corresponds to 
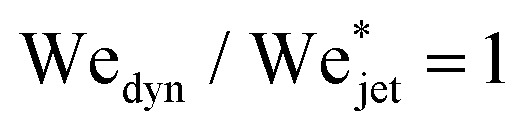
 and the dashed line is 
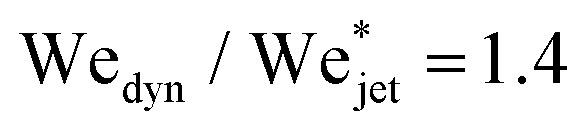
 obtained by averaging the minimum value of observed traversing for all the liquids (excluding the AOT solutions). The experimental data are in good agreement with the model, *i.e.*, for each liquid, most of the open symbols lie above the dashed line and conversely for closed symbols. Uncertainty was calculated for all the experimental data and example error bars are shown at selected points, where the uncertainty was found to increase linearly with the rescaled Weber number.

From Fig. 6, we observe that most of the open symbols lie above the same approximate value of ∼1.4 and conversely for closed symbols. The exception is formed by the data for the AOT solutions, where it is possible that *γ*_dyn_ is underestimated as *γ*_dyn_ ≈ 32.2 mN m^−1^, and in fact lies closer to the surface tension of water of 72.1 mN m^−1^. An accurate measurement of the dynamic surface tension in such timescales is challenging^29,34^ and is out of the scope of this work. However, as demonstrated by our results, the dynamic surface tension can play a pronounced role in different dynamic conditions. Therefore, we can safely conclude that the impact process is initially dominated by inertia and that the surface tension is the major opposing force.

**Fig. 7:**
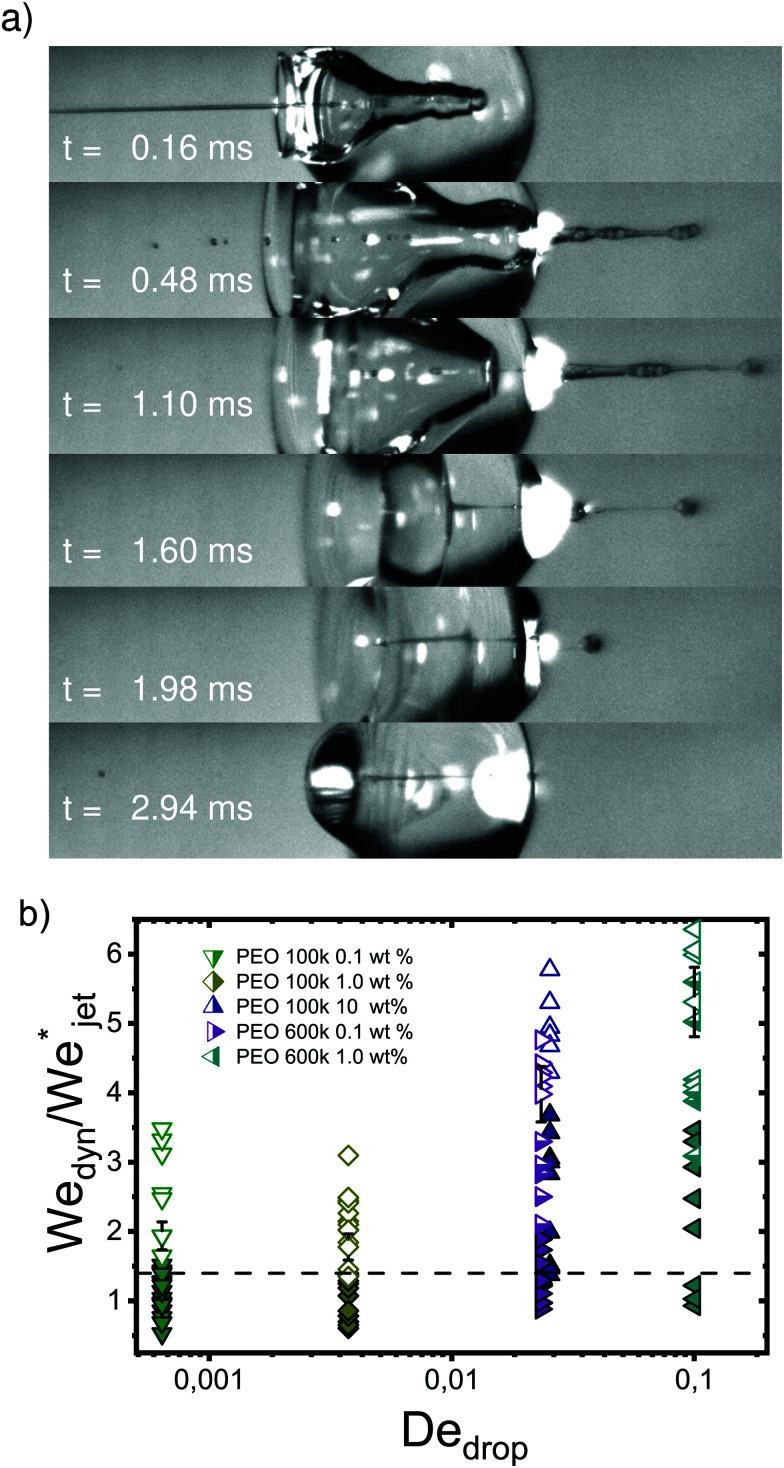
(a) Snapshots of a liquid jet impacting a water droplet with PEO 600k 1 wt% showing pearling (see Movie S5 in the ESI†). A jet with *U*_jet_ = 33.5 m s^−1^ and We_jet_ = 1160 impacts a droplet with *D*_drop_ = 2.21 mm. The jet travels a distance larger than *D*_drop_, but due to the viscoelastic effects of the droplet, it gets sucked back into the droplet. (b) Phase diagram in terms of De and 
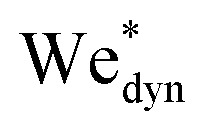
. Open symbols represent cases where the jet traverses the droplet, solid ones stand for the embedding case and half filled ones represent pearling. The dashed line represents the transition from traversing to embedding cases observed for Newtonian liquids. Uncertainty was calculated for all the experimental data and example error bars are shown at selected points, where the uncertainty was found to increase linearly with 
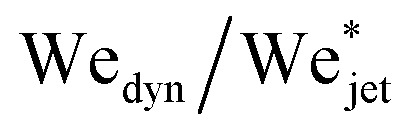
.

Turning to the viscoelastic droplets, Fig. 7b shows data for the jet traversing and embedding impact cases for droplets consisting of the PEO solutions. In this figure, we plot 
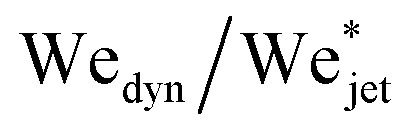
 against the Deborah number defined as De = *λ*/*τ*_c_, where *λ* is the relaxation time of the polymer (see Table 1) and *τ*_c_ = (*ρ*_drop_*D*_drop_^3^/*γ*_drop_)^1/2^ is the capillary timescale. We use this definition of the Deborah number for our PEO solution droplets, as we expect to observe deviations from the Newtonian behaviour when *λ* becomes comparable to the scale at which surface tension starts to influence the cavity dynamics, *i.e.*, at the capillary time scale *τ*_c_. In Fig. 7, open and closed symbols again represent traversing and embedding cases respectively and half-filled symbols denote an intermediate state between traversing and embedding, which we call pearling. During pearling, the jet travels a distance larger than *D*_drop_ after impact and thus protrudes from the droplet, but due to the viscoelastic properties of the liquid it gets sucked back into the droplet, as visualised in the experimental snapshots in Fig. 7a.

From Fig. 7b it is observed that the traversing and embedding process for the PEO solutions with De ≲ 4 × 10^−3^ is similar for Newtonian liquids, leading to the same threshold value 
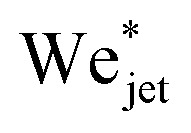
, showing that the viscoelastic effects are weak. However, as De increases, *i.e.*, when the viscoelastic timescales become increasingly comparable to the capillary time, the jet needs larger speeds to traverse the droplet. This is in line with previous experiments where by increasing the elastic modulus of gelatin the cavity depth of an impacting sphere would decrease, keeping the impact velocity constant.^35^ These results show that viscoelastic properties as described by De significantly change the traversing dynamics. This is crucial information when trying to understand needle-free injections on skin, as it has been shown that skin has viscoelastic properties.^36^ However, conducting systematic studies trying to quantify the influence of skin properties during injection processes is challenging, because of high variability from person to person and even between different parts of the body.^36,37^ Furthermore, studying the viscoelastic properties of skin is in itself challenging given the opacity of skin.^38,39^ In this context, our results present information about the characteristics of the impact with a simpler system than skin, isolating the effects of individual material properties of the target from the enormous complexity of skin.

### 4.2 Cavity dynamics

**Fig. 8:**
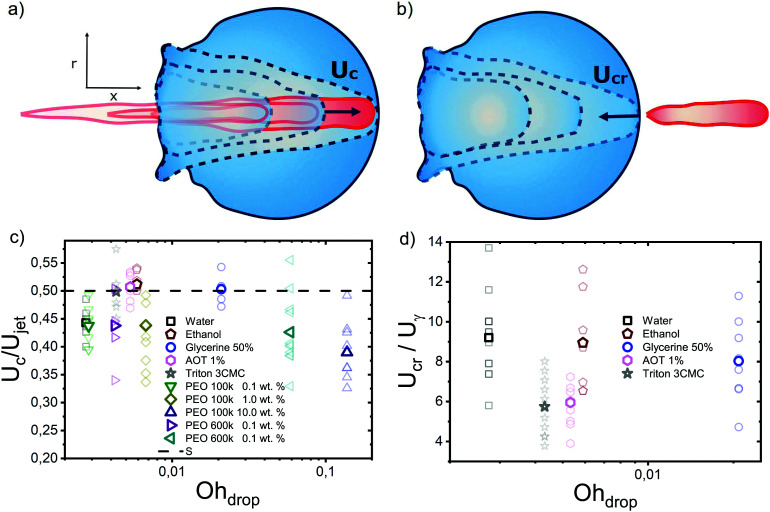
Sketch illustrating the definitions of (a) the cavity velocity *U*_c_ and (b) the velocity of the retracting cavity *U*_cr_. (c) Ratio of the cavity velocity *U*_c_ and the impact velocity *U*_jet_, compared to the expected value of 0.5. (d) *U*_cr_ divided by the capillary velocity scale 
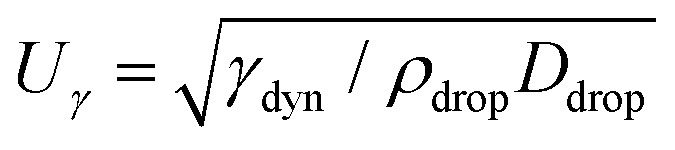
; average values for each liquid are statistically similar, indicating that the retraction of the cavity is governed by capillary forces. For each liquid, bold coloured symbols represent average values of light symbols.

To obtain more insight into the dynamics of the cavity that is created in the droplet, we studied the cavity velocity in the positive *x* direction, *i.e.*, while the jet is penetrating into the droplet, as sketched in Fig. 8a. For each liquid we plot the average ratio of the cavity velocity *U*_c_ and the jet velocity *U*_jet_ (bold symbols), together with the values obtained for each individual experiment (light symbols) as a function of Oh_drop_ in Fig. 8c. The measured and averaged values are remarkably close to the value *U*_c_/*U*_jet_ = 0.5, which is to be expected for the impact of a continuous jet on a pool, and is in agreement with previous works.^2^ The slight deviation observed for the water and the PEO solutions droplets could be due to water and PEO solution droplets being the largest ones used in the experiments. In that case, the breakup of the jet could influence *U*_c_, similarly to a train of droplets impacting a deep pool.^8,9^

In addition to *U*_c_, we measured the retraction cavity velocity *U*_cr_ after the cavity reached its maximum length, as sketched in Fig. 7b. In Fig. 8d, we show *U*_cr_ rescaled by the capillary velocity scale 
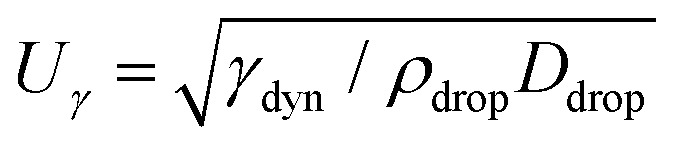
. We observe that the average of data for the different liquids is similar, taking into consideration the data dispersion. The average of ethanol, water and aqueous glycerol mixtures is even statistically indistinguishable, given the error margins of the experiment. The lower average values of *U*_cr_ for the AOT and Triton solutions can possibly be explained by the Marangoni stresses generated by the flow from areas with low surface tension to those with high surface tension. Indeed, Marangoni stresses have been shown to retard cavity collapse and slow the velocity of Worthington jets.^40,41^ Therefore, we can assume that *U*_cr_ ∝ *U*_*γ*_, indicating that the retraction of the cavity is surface tension driven.^42^ The origin of the dispersion in *U*_cr_ is associated with the jet tail breakup, where the Matryoshka effect or the creation of an antibubble may arise, like in Fig. 5.^28,29^

### 4.3 Observations after the cavity collapse

After the retraction phase, the cavity collapses and generates a Worthington jet (as *e.g.*, depicted in the last panel of Fig. 3). Extensive studies of the length, speed and breakup time of a Worthington jet formed after droplet and solid impact on a liquid pool have been widely reported, and are outside of the scope of this paper.^1,40,42–47^ Moreover, given the random breakup of the impacting jet in our experiments, the Worthington jets are observed to vary widely in size and shape, even when the droplet and impacting jet consist of the same liquids. This is understandable, as it has been shown in the literature that small disturbances in the cavity can have a strong influence on the Worthington jet properties.^42^

**Fig. 9:**
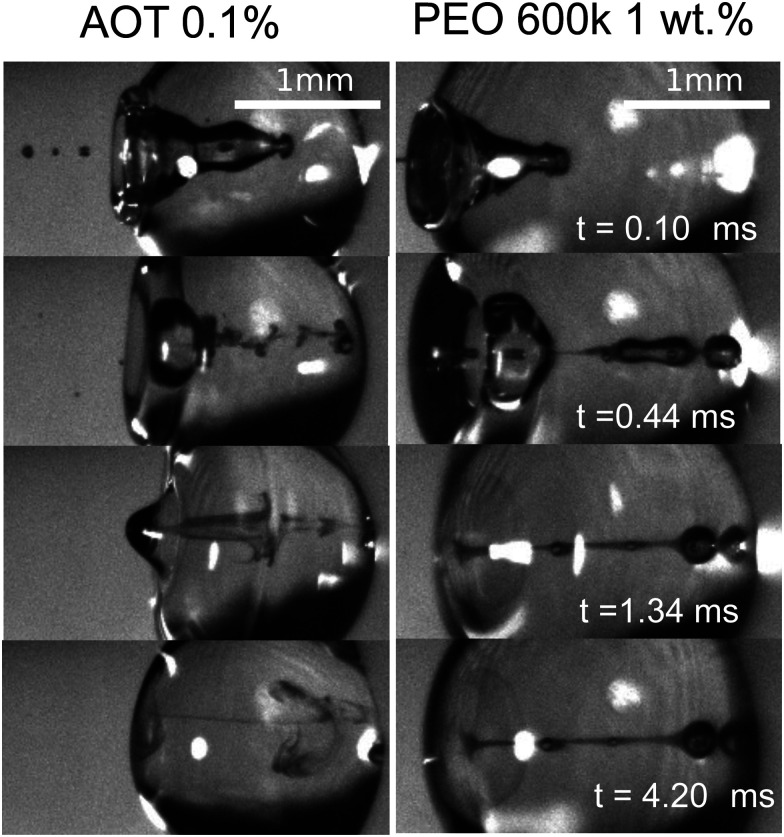
Mixing after impact and cavity collapse. Left, jet impacting an AOT 0.1 wt% droplet with We_jet_ = 359. In the sequence we observe vortical mixing presumably due to the Marangoni flow caused by the difference of surface tension between the jet and the droplet (see Movie S6 in the ESI† and Jia *et al.* 2020^40^). Right, jet impacting a PEO 600k 1 wt% with We_jet_ = 574. In this sequence we observe little mixing and diffusion due to the viscoelastic properties of the droplet even after 0.42 ms.

Lastly, we observe that the mixing and diffusion of the impacting jet into the droplet is governed by the droplet characteristics. Indeed, for a jet impacting a water droplet with AOT 0.1 wt% below the critical value 
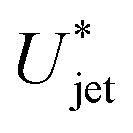
 needed for traversing, there is vortical mixing (Fig. 9, left). Comparable mixing patterns were observed (data not shown) for the rest of the Newtonian liquids containing surfactants, and weaker mixing is seen for water, ethanol and glycerine 25% droplets. Similar mixing patterns have been described in the literature, for example, Jia *et al.* in 2020^40^ reported an interfacial Marangoni flow enhancing the mixing of an impacting droplet and a liquid pool with different surface tensions.

We note that in our experiments the surface tension of the jet is almost always expected to be different from the surface tension of the droplet, and a Marangoni flow could explain this type of mixing. However, a more in depth study is needed to confirm this hypothesis. In contrast, for the viscoelastic liquids with De ≳ 2 × 10^−2^ and the glycerol mixture liquids with Oh ≳ 0.02, the jet does not mix with the droplet in the timescale of our experiments (Fig. 9, right). Furthermore, low viscosity (Oh ≲ 0.01) and low surface tension liquids reach equilibrium at a later stage than more viscous liquids (Oh ≳ 0.02) and with higher surface tension. For example, in Fig. 9 a PEO 600k 1 wt% droplet reaches equilibrium ∼4 times faster than the AOT droplet. This is expected, as surface tension and viscosity have been observed to affect droplet oscillations.^48,49^

## 5 Conclusions

We have presented experimental results of liquid water jets impacting on pendant droplets with different liquid properties. We proposed two models to predict a critical jet impact velocity beyond which the jet traverses the droplet. First, we presented a model based on a simple energy balance between the jet kinetic energy and the change in surface tension of the droplet. The second model is based on the comparison between the Young–Laplace and the dynamic pressures of the cavity made by the penetrating jet, and its shape is described by the two-dimensional Rayleigh equation.

Although the critical velocity predicted in both models is of the same order of magnitude, they differ in their scaling relation with *D*_drop_/*D*_jet_. The difference arises from the different description of the cavity geometry and its associated surface energy. In the energy balance model, a cylindrical shape is assumed, contrasting with the more accurate cavity shape described by the two-dimensional Rayleigh equation. Furthermore, we tested the validity of the second model, by fitting our experimental data with eqn (11), showing good agreement when dynamic surface tension effects are considered, see Fig. 6. Therefore, for Newtonian droplets the impact process is initially dominated by inertia and their dynamic surface tension is the major opposing force.

In addition, we investigated viscoelastic effects by using water-based polyethylene-oxide solutions of varied concentrations and molecular weight. For De ≲ 4 × 10^−3^, the droplets act as if they were Newtonian. In contrast, for De ≳ 2 × 10^−2^, a greater jet impact speed is necessary to traverse the droplet, indicating that when the capillary and relaxation times are comparable, viscoelastic effects can dominate the traversing phenomena. Moreover, we observed a distinct transition phenomenon from traversing to embedding, which we called pearling and during which the protruding jet is sucked back into the droplet.

Next, we investigated the advancing and retraction velocities *U*_c_ and *U*_cr_ of the cavity, confirming previous reports that *U*_c_/*U*_jet_ ∼ 0.5 for different liquids. Furthermore, we found that *U*_cr_ is surface tension driven, with the connotation that for droplets containing surfactants *U*_cr_ is observed to be slower than for the other liquids that were used, which could be explained by Marangoni stresses.

Our results are relevant for needle-free injections into soft tissues such as skin, or the eyes, where controlling the jet velocity, 
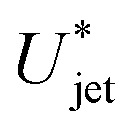
, would be essential to avoid undesired tissue damage and ensure successful drug delivery.

## Author contributions

M. A. Q. S., D. v. d. M. and D. F. R. conceived the experiments, analysed the results and wrote the manuscript. M. A. Q. S. and D. F. R. designed and built the experimental setup. M. A. Q. S. performed the experiments. I. W. H. participated in the discussions and wrote the manuscript.

## Conflicts of interest

There are no conflicts to declare.

## Supplementary Material

SM-017-D1SM00706H-s001

SM-017-D1SM00706H-s002

SM-017-D1SM00706H-s003

SM-017-D1SM00706H-s004

SM-017-D1SM00706H-s005

SM-017-D1SM00706H-s006
